# Cell Penetrating Peptides Used in Delivery of Therapeutic Oligonucleotides Targeting Hepatitis B Virus

**DOI:** 10.3390/ph13120483

**Published:** 2020-12-21

**Authors:** Bénédicte Ndeboko, Serge Thierry Omouessi, Brice Ongali, Augustin Mouinga-Ondémé

**Affiliations:** 1 Département de Biologie Cellulaire and Moléculaire-Génétique, Faculté de Médecine, Université des Sciences de la Santé, Libreville 241, Gabon; ongali.brice@gmail.com; 2 Département de Physiologie, Faculté de Médecine, Université des Sciences de la Santé, Libreville 241, Gabon; omouessi@hotmail.fr; 3 Centre Interdisciplinaire de Recherches Médicales de Franceville, Unité des Infections Rétrovirales et Pathologies Associées, Franceville 241, Gabon; ondeme@yahoo.fr

**Keywords:** hepatitis B virus (HBV), Peptide Nucleic Acids (PNAs), small interfering RNAs (siRNAs), Cell Penetrating Peptides (CPPs), drug delivery, duck hepatitis B virus (DHBV), antiviral therapy

## Abstract

Peptide Nucleic Acid (PNAs) and small noncoding RNAs including small interfering RNAs (siRNAs) represent a new class of oligonucleotides considered as an alternative therapeutic strategy in the chronic hepatitis B treatment. Indeed, chronic hepatitis B virus (HBV) infection remains a major public health problem worldwide, despite the availability of an effective prophylactic vaccine. Current therapeutic approaches approved for chronic HBV treatment are pegylated-interferon alpha (IFN)-α and nucleos(t)ide analogues (NAs). Both therapies do not completely eradicate viral infection and promote severe side effects. In this context, the development of new effective treatments is imperative. This review focuses on antiviral activity of both PNAs and siRNAs targeting hepatitis B virus. Thus, we briefly present our results on the ability of PNAs to decrease hepadnaviral replication in duck hepatitis B virus (DHBV) model. Interestingly, other oligonucleotides as siRNAs could significantly inhibit HBV antigen expression in transient replicative cell culture. Because the application of these oligonucleotides as new antiviral drugs has been hampered by their poor intracellular bioavailability, we also discuss the benefits of their coupling to different molecules such as the cell penetrating peptides (CPPs), which were used as vehicles to deliver therapeutic agents into the cells.

## 1. Introduction

Hepatitis B virus (HBV) infection remains a significant public health problem, despite the availability of an effective prophylactic vaccine. Currently, over two billion people have been infected in the world, including 240 million chronic HBV carriers [1]. Moreover, these chronic HBV patients are at high risk of developing severe liver diseases such as cirrhosis and hepatocellular carcinoma (HCC), which account for 600,000 deaths per year [2].

HBV is a prototype member of the hepadnavirus family, which contains a partially double-stranded relaxed circular DNA (rcDNA) and replicates through an RNA intermediate. Indeed, after infection, HBV replication takes place in nuclei of infected hepatocytes. Subsequently, rcDNA is repaired by host cellular polymerases to a covalently closed circular DNA (cccDNA), termed viral minichromosome. The cccDNA is the template for viral transcription, including viral messenger RNA (mRNA) and viral pregenomic RNA (pgRNA) synthesis [3]. Moreover, replication of HBV genome depends on pgRNA, reverse transcriptase (RT), and takes place through a complex mechanism connecting primer shifting which is facilitated by 3-terminal direct repeat (DR) sequence of pgRNA. Indeed, DR sequence is a crucial functional element in viral synthesis, because it serve as the primary sites for viral replication. Importantly, six overlapping open reading frames (ORFs) are identified and four indicated (i) the preS/S ORF encodes three hepatitis B surface antigens (HBsAg), termed large (L), middle (M) and small (S). (ii) The precore/core ORF, encodes the core protein (HBcAg) and a non-structural protein termed precore (HBeAg). (iii) The pol ORF encodes viral polymerase. (iv) The X ORF, encodes a small regulatory protein called x protein (HBx) [4].

Chronic HBV infection occurs due to the persistence of cccDNA inside infected hepatocytes, because current therapeutics such as pegylated interferon-a and nucleos(t)ide analogs (NUCs) only decrease serum viral load to an undetectable level, but they cannot completely eradicate the viral cccDNA. Furthermore, long-term treatment with these therapeutic agents is expensive and is associated with severe side effects. Therefore, alternative treatment option for patients chronically infected with HBV need to be urgently developed [5,6].

In this regard, a new class of oligonucleotides such as Peptide Nucleic Acid (PNA) and small noncoding RNAs (siRNAs) represent an alternative therapeutic strategy in the chronic hepatitis B treatment. Indeed, PNAs are a class of synthetic DNA mimics in which sugar-phosphate backbone is replaced by *N*-2-aminoethylglycine repeating units [7]. PNAs have the capacity to hybridize with high affinity and sequence specificity to complementary DNA or RNA sequence through the Watson-Crick base pairing.

Furthermore, PNAs are particularly stable into the cells because they are able to resist to degradation by nucleases and proteases [8]. These characteristics make PNAs molecules promising candidates for clinical applications in therapeutic approach of HBV chronic infection. Interestingly, we have previously demonstrated in the Duck Hepatitis B virus (DHBV), infection model that antisense PNA targeting the HBV signal ε can inhibit viral reverse transcription in rabbit reticulocyte lysate system [9]. Indeed, the DHBV model is a reference for evaluation of novel anti-HBV approaches. Due to the extremely narrow host range of HBV that infects only humans and chimpanzee, the closely related duck HBV (DHBV) provides a unique opportunity to study the antiviral potential of drugs in vitro, in primary duck hepatocyte culture (PDH) and in vivo in Pekin ducklings.

Nevertheless, the application of the potential of PNAs as antiviral drugs in therapeutic approach has been hampered by their poor intracellular uptake. Indeed as a large hydrophilic molecule, PNAs do not cross lipid membranes easily. Thus, a series of cellular delivery system have been developed during the last few years including cell penetrating peptide (CPPs) to enhance intracellular internalization.

The CPPs are also called membrane translocation sequences (MTSs) or protein transduction domains (PTDs). These molecules are a class of diverse short cationic peptides of a few dozen amino acids, which are able to translocate though various biological membranes [10,11]. Therefore, CPPs can efficiently internalize a wide range of different bioactive molecules into the cells, such as PNAs and siRNA [12,13].

Interestingly, the first CPP termed Tat, was found within the human Immunodeficiency virus (HIV) transactivating regulatory protein, [14,15]. A few years later, Penetratin, the third helix of Drosophila Antennapedia homeodomain protein (pAntp) [16,17] and others CPPs including VP22, a herpes virus protein [18], Transportan, a 27 amino acid chimeric peptide [19] were discovered. Remarkably, several synthetic CPPs such as polyarginine and polylysine have been developed [12,20,21]. Recently, a novel CPP, termed X-pep, from X-protein of HBV has been identified [22]. Taken together, these CPPs can cross cell membrane and efficiently deliver various cargoes as PNAs and siRNAs into the cell.

The small interfering RNAs (siRNAs) are the small noncoding RNAs which are artificial double stranded RNAs of 19–21 nucleotides in length [23]. RNA interference is an endogenous process in which siRNAs post-transcriptionally regulate gene expressions [24]. Because of its exceptional roles in regulating the functions and stabilities of mRNAs, siRNA has emerged as a promising alternative therapeutic approach for the treatment of viral diseases including chronic HBV infection [25,26,27,28]. However, the synthetic siRNAs specifically targeting HBV need to be delivered into the hepatocytes to silence viral genes. Indeed, despite RNA interference being a very efficient mechanism in gene silencing, siRNA are poorly transported across cell membrane as naked molecules. Thus, the CPPs have been used widely as carriers to deliver siRNAs in vitro and in vivo [29,30].

Taken together, antisense PNAs and siRNAs are two promising approaches for inhibiting specific HBV genes. In this report, we focus on antiviral activity of both PNAs and siRNAs targeting hepatitis B virus. Therefore, we briefly present our results on the ability of PNAs to inhibit hepadnaviral replication in Duck Hepatitis B Virus (DHBV) model. We also discuss the role and the benefit of CPPs as carriers in increasing uptake efficacy of a both PNAs and siRNA. Moreover, antiviral activity of CPPs used as vehicles will be documented in this review.

## 2. Antiviral Activity of PNAs Alone on Hepatitis B Virus

Because current drugs approved for chronic hepatitis B treatment only target viral polymerase and show a virostatic effect since rebound of HBV replication is common after treatment cessation [31], we have evaluated the therapeutic potential of PNAs as an alternative antiviral approach. In the DHBV infection model, we have investigated the ability of PNAs alone targeting epsilon (ε), the hepadnavirus encapsidation signal, which plays an important role in the initiation of viral reverse transcription (RT) [32,33,34] to inhibit viral replication.

To further explore antiviral effect of this anti-ε PNA alone, firstly, our team has used a cell-free system of enzymatically active DHBV RT expression. To measure anti-ε PNA alone activity, a quantitative dot blot assay was performed after the RT reaction and radioactivity was measured in a scintillation counter [9]. Our results showed that PNAs targeting (ε), exhibited a strong antiviral effect at low nanomolar concentrations on DHBV RT, in a sequence-specific manner [9]. Interestingly, anti-ε PNA induced a decrease in a dose-dependent manner of DHBV reverse transcription. Importantly, PNA 2053 (H-gca-atg-tag-acg-taa-Lys-NH2) appeared to be more efficient (>90% of inhibition) than PNA 2052 (H-tag-acg-taa-aga-tac-Lys-NH2) on RT elongation reaction. This difference in activity of these PNAs could be explained by their structure. Indeed, PNA 2053 is complementary not only to the unpaired bulge, as PNA 2052, but also to the entire epsilon upper stem.

Moreover, the analogous S-ODNs have no or poor inhibitory effect on the viral RT [9]. To further determine the inhibition specificity, a 2-base mismatch PNA and S-ODN were tested, the results showed that the control PNA showed no marked inhibitory effect on DHBV RT, indicating very high specificity of the PNAs.

In contrast, the analogous S-ODNs showed a much lower mismatch discrimination than the PNAs. Altogether, our results demonstrate that the anti-ε PNA alone strongly and specifically inhibit DHBV reverse transcription at very low concentrations at which the analogous S-ODNs resulted in neither the marked decrease of viral RT in cell-free system of enzymatically active DHBV RT expression. To investigate if RT activity inhibition exhibit by PNAs was due to an effect on the initiation step, the RT reaction was performed in presence of anti-ε PNA alone and the results showed the strong inhibition of both RT initiation and elongation when anti-ε PNA alone was added simultaneously with the template RNA.

Interestingly, the RT inhibition by anti-ε PNA alone was time-dependent indeed, addition of anti-ε PNA alone at the beginning of the DHBV polymerase expression led to 95% inhibition of reverse transcription reaction while the same anti-ε PNA alone added at the end of the reaction inhibited the RT elongation by only 37% [9]. Taken together, these results suggest that PNAs act at early stage of DHBV reverse transcription mechanism. Next, the inhibitory effect of anti-ε PNA alone was tested in in vitro in primary duck hepatocyte cultures (PDHs), which were infected with DHBV. The HBV elimination was measured by a molecular assay. Indeed, the DNA was extracted from hepatocyte cells and was analyzed by Southern blot using a 32P-labelled DHBV probe as described previously described [12]. Viral DNA was quantified by PhosphorImager using ImageQuant software (Molecular Dynamics) [9,12]. The results showed that anti-ε PNA alone decreased viral DNA by 30% as compared with the untreated controls PDH. Importantly, this inhibition induced by anti-ε PNA alone was specific since the PNA control showed no significant inhibition on intracellular DHBV replication (Table 1) [9].

Otherwise, our in vivo results showed that treatment of DHBV-infected ducklings by anti-ε PNA alone, decrease viremia and intrahepatic viral replication, compared with the untreated animal controls (data not shown).

## 3. Anti-HBV Effect of siRNA Alone

Because Hepadnavirus replication requires a key step of reverse transcription for synthesis of pregenomic RNA, using siRNA targeting HBV genes could lead to inhibition of viral replication (Table 1). In this context, Zhang et al. in their study, demonstrated that siRNA could significantly decrease HBsAg and HBeAg antigen expression into Huh-7 cells [25]. Importantly, among all the siRNA used, pSi-HBV1 which targeting a both S and P HBV genes was most effective. Indeed, pSi-HBV1 led to a decrease in HBsAg and HBeAg antigens expression by 86% and 83% respectively, 72 h after transfection [25]. Moreover, the siRNAs specifically targeting HBV RNA such as pSi-HBV1 and pSi-HBV2 led to reduction of all the viral transcripts by 90% [25].

Otherwise, in other studies, the potential of RNAi application in anti-HBV therapeutic approach was evidently demonstrated. Indeed, RNA interference target HBV DR elements and regions that code for core, polymerase, PreS, S, and X proteins could efficiency inhibit HBV replication. Interestingly, the delivery of several RNAi lead to strong synergistic antiviral effect in vivo, in a hydrodynamic transgenic mice model [35]. Moreover, Wu et al. indicated that a dual siRNA expression system, could instantaneously express two different siRNA molecules which can explicitly target the HBs and HBx genes of HBV, respectively, in Bel-7402 and HepG2.2.15 cells. The results showed that dual siRNA could simultaneously inhibit the expression of HBs and HBx gene by 83.7% and 87.5%, respectively [36].

## 4. Benefit of CPPs Used as Vehicles in Oligonucleotides Delivery

The antiviral efficiency of several oligonucleotides as PNAs and siRNA can be improved by their coupling to CPPs used as vehicles. Indeed, our studies showed that CPPs considerably increased PNAs uptake into the hepatocytes in vitro in primary duck hepatocyte cells and in vivo in duckling [9,11,12,37]. Otherwise, other studies showed the benefit of siRNA coupling to CPPs on their intracellular delivery [38,39] and on their antiviral activity [28,40].

### 4.1. Inhibitory Effect of CPP-PNA Conjugates on Hepadnavirus Replication

**Figure 1:**
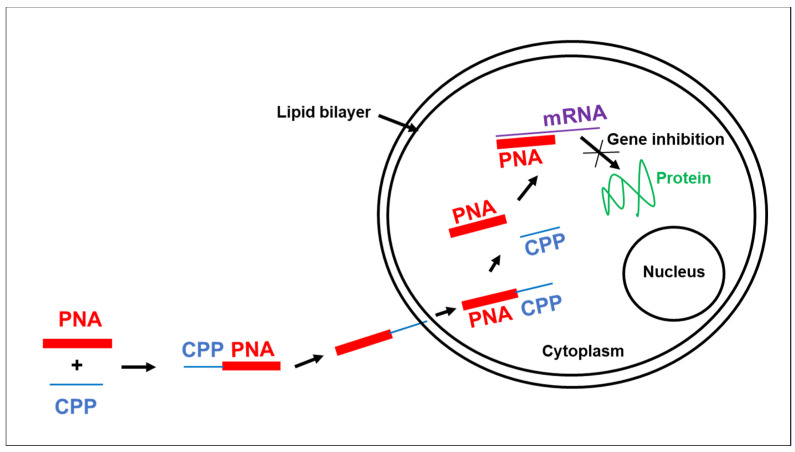
The coupling of Peptide Nucleic Acid (PNA) to cell penetrating peptides (CPP), a nonviral gene delivery system, improves their intracellular uptake and induces gene inhibition.

Because the main difficulty in the use of PNAs as antiviral drugs is their poor transport across the lipid bilayer membrane and intracellular distribution, we conjugated the anti-ε PNA to a cationic Cell Penetrating Peptide (CPP) (Figure 1). In primary duck hepatocytes (PDH) cultures infected by DHBV, we evaluated antiviral effect of anti-ε PNA coupled to polyArginine ((Arg)_7_) or to nuclear localization signal (NLS) CPP. The results showed that anti-ε PNA coupled to polyArginine ((Arg)_7_) exhibit a strong inhibitory effect [9]. Indeed, the treatment of DHBV-infected PDH by this CPP-PNA lead to a decrease by 65% of total intracellular viral DNA as compared with the untreated control cells. Interestingly, polyArginine ((Arg)_7_) strongly enhanced the inhibitory effect compared to anti-ε PNA alone, which only decreases DHBV replication by 30% [9]. In addition, anti-ε PNA conjugated to NLS peptide did not enhance the inhibitory effect since it leads to a similar inhibition than exhibit by anti-ε PNA alone (30%). Importantly, the quantitative analysis of single stranded DNA (ssDNA), which is a product of viral reverse transcription reaction, showed a similar inhibition (65%) in the amount of DHBV DNA in anti-ε ((Arg)_7_)-PNA treated cells as compared to untreated cell controls [9]. This viral inhibition by anti-ε ((Arg)_7_)-PNA was specific because a 4-nt or 2-nt mismatched PNA coupled to the same CPP peptide showed no marked inhibitory effect on intracellular viral replication. Moreover, neither anti-ε ((Arg)_7_)-PNA conjugated nor its corresponding mismatch exhibited toxicity, in vitro, in PDH cultures. In another study, we used the same DHBV infection model and the same anti-ε PNA conjugated to different CPPs such as (D-Arg)_8_ and (D-Lys)_4_ to evaluated the role of these CPPs in intracellular delivery of this PNA targeting DHBV replication. Our results showed that the anti-ε PNA conjugated to (D-Arg)_8_ inhibits DHBV replication in vitro in PDH and in vivo in ducklings, compared with the untreated PDH or duckling controls [12]. Surprisingly, a similar inhibition of DHBV replication was observed in vitro in PDH and in vivo in ducklings for a 2-nt mismatched PNA control coupled to (D-Arg)_8_ peptide. Because the treatment with a 2-nt mismatched PNA control coupled to (D-Arg)_8_ peptide led to a decrease of viral replication, we tested the antiviral effect of (D-Arg)_8_ peptide alone in vitro in PDH and in vivo in ducklings in the similar experiments. Remarkably, the treatment of infected PDH or ducklings with (D-Arg)_8_ peptide alone induced inhibition of DHBV replication a both in vitro in PDH and in vivo in ducklings [12]. Interestingly, the same anti-ε PNA conjugated to (D-Lys)_4_ inhibited viral replication in a sequence-specific manner since (D-Lys)_4_ alone did not display antiviral activity in the absence of anti-ε PNA cargo. Our results provide the first evidence that CPP-PNA conjugates inhibit Hepadnavirus replication in vitro and in vivo without toxicity. Importantly, these results established the key role of CPP used as vehicles in antiviral specificity of CPP-PNA conjugated. Highly, the antiviral activity of all these molecules was not associated with toxicity (Table 1) [12].

### 4.2. CPP-siRNA Conjugates Decrease Viral Replication

**Figure 2:**
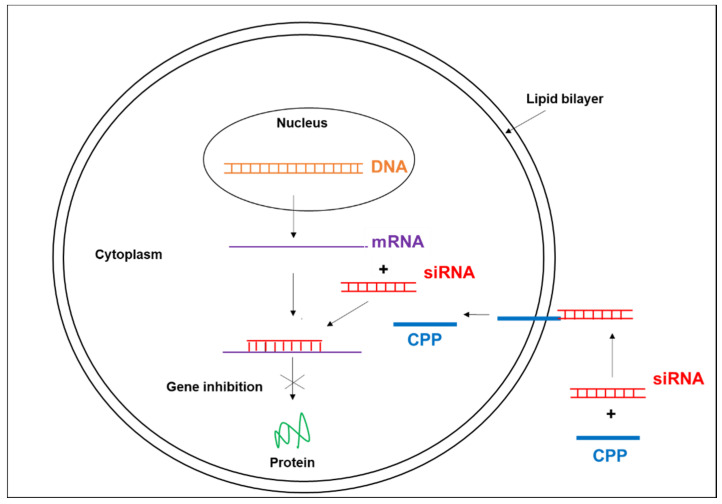
The CPP, a nonviral gene delivery system, favors intracellular uptake of small interfering RNA (siRNA) and induces gene inhibition.

The small interfering RNAs (siRNAs), are a promising approach for gene therapy, however their intracellular administration remains difficult. To date, a great variety of delivery system for siRNAs has been investigated such as lipids, polymers, aptamers and cell penetrating peptides (CPPs) [26,27,28,29,30,31,32,33,34,35,36,37,38,39,40,41,42,43,44]. In this regard, CPPs appear as a new class of transporters, which increase intracellular uptake of biologically active molecules including siRNAs [45] (Figure 2). Concerning hepatitis B virus infection, few or no studies are available currently on CPP-siRNAs targeting HBV. Thus, to enhance intracellular uptake of siRNA targeting conserved HBV sequences in the recent study, a modified peptide was co-administrated with this siRNA sequence which was conjugated to cholesterol. The results indicated an inhibitory effect of viral DNA, viral RNA, and proteins in transgenic mouse model of HBV infection (Table 1) [28].

Importantly, the electrostatic interactions between positively charged amine groups of the polycations and negatively charged phosphate groups of the siRNA lead to complexes formation, which interact with negative charges of cell membrane. These interactions favor intracellular uptake of these complexes, thus entry of siRNAs. In addition, siRNAs can be coupled to other compounds and reduce HBV replication in hepatocytes compared with that achieved with uncoupled siRNAs [46,47].

Otherwise, combination of siRNA with the current drugs as lamivudine (3TC) may display strong inhibitory effect on HBV replication. Indeed, the simultaneous treatment of HepG2.2.15 cells with siRNA and lamivudine showed the decrease of HBV replication [40]. Interestingly, in several studies, CPPs were used as siRNAs delivery system and this coupling improved their biological activities [13,48].

### 4.3. CPP Alone as Potential Anti-HBV Drugs

Currently, the biological activities of CPPs have been reported including antiviral activities [48]. Moreover, the antiviral activity of CPPs has been more studied for enveloped viruses such as Herpes Simplex Virus (HSV) and Human Immunodeficiency virus (HIV) [49,50,51,52]. Indeed, few or no studies which investigated the ability of CPPs to inhibit HBV replication are available. In order to find an efficient anti-HBV treatment, we have evaluated several molecules such as PNA alone or conjugated to CPPs. Surprisingly, we have discovered that some of these CPPs exhibited anti-HBV activity in the absence of their PNA cargo. Thus, the treatment of DHBV-infected PDH or DHBV-infected ducklings by (D-Arg)_8_ peptide decrease Hepadnavirus replication in vitro and in vivo, in duck HBV (DHBV) model (Table 1) [12]. Hence, this antiviral activity of (D-Arg)_8_ alone may affect the sequence-specificity of CPP-PNA conjugates [12]. In addition, using the same DHBV infection model, we have investigated the ability of the lipid modified CPPs peptide such as Decanoyl-(D-Arg)_8_ to inhibit Hepadnavirus replication in vitro in stably DHBV-transfected chicken hepatoma cells (LMH-D2) and in PDH cultures. Our results showed that the treatment of these cells with different concentrations of Decanoyl-(D-Arg)_8_ led to a strong and dose-dependent decrease of viral replication. Indeed, Decanoyl-(D-Arg)_8_, drasticaly inhibit the late stages of viral replication (Table 1). Importantly, this inhibition was greater than that obtained with (D-Arg)_8_ indicating that the antiviral effect exhibited by (D-Arg)_8_ can be improved by the amphipathic molecule used to modify this CPP. Interestingly, these results can be extend to human virus, because the treatment of the stable HBV-transfected cell line, HepG2.2.15, which constitutively secretes HBV by Decanoyl-(D-Arg)_8_ led to a dose-dependent inhibition of HBV secretion [53]. In another study, several artificial recombinant peptides comprising arginine 7 were used for treatment of HepG2.2.15 cells. The results showed that this CPP can efficiently enter into HepG2.2.15 and cause obstruction of the nucleocapsid assembly and inhibit HBV release [54].

## 5. Effect of These Oligonucleotides on Others Viruses

To date, several studies have shown the antiviral potential of some oligonucleotides on various virus including HIV, HCV, SARS-coronavirus. Indeed, Human immunodeficiency virus type 1 (HIV-1) is the first virus shown to be inhibited by siRNA when these molecules target various viral regions [54]. In addition, numerous regions of HCV’s RNA genome are sensitive to the action of siRNA and PNAs [55,56,57].

Otherwise, severe acute respiratory syndrome (SARS) was caused by SARS-associated coronavirus (SARS-Cov). Currently, a new coronavirus is responsible of a pandemic that affects all countries in the world. SARS-Cov is a large enveloped, positive-stranded RNA virus and its genome is composed of several genes: replicase (rep), spike (S), envelope (E), membrane (M), and nucleocapside (N). Because there are no treatment and vaccines against SARS-Cov infection, the ability of new drugs such as siRNA to inhibit viral replication were tested. Thus, siRNA targeting *rep* gene showed the remarkable inhibition of SARS-Cov replication [58]. These results greatly encouraged the clinical trials of siRNAs as an anti- SARS-Cov therapy.

## 6. Other Oligonucleotide Delivery Systems

Currently, many other non-viral delivery systems of oligonucleotides are used. Indeed, to improve their intracellular uptake, the oligonucleotides can to be conjugated to sugars, lipids, polymers, aptamers, etc. Thus, the therapeutical potential of these oligonucleotides conjugates is increased.

### 6.1. Sugar-Oligonucleotide Conjugates

The oligonucleotides can to be associated covalently with several sugars such as Galactose alone or Lactose. Certainly, the synthesis of these carbohydrate-conjugates has been documented [37,59] and the results showed that the coupling of these oligonucleotides with carbohydrates enhance their entry into the hepatocyte cell and their stability. In addition, the asialoglycoproteins receptors (ASGP-R), which are found on the hepatocyte cell membranes, were the first mammalian lectins defined. They play an essential role in the hepatocellular uptake, through receptor-mediated endocytosis mechanism, of several biological molecules having a galactose-terminal carbohydrate in their structure [30,59]. Consequently, carbohydrate-siRNA conjugates exhibit a strong gene silencing levels into the liver both in vitro, in hepatoma cell lines and in vivo, in murine models [59]. Moreover, we evaluated the anti-HBV activity of sugar-modified CPP-PNA conjugates in the HepaRG human hepatoma cell line and our results demonstrated that treatment of HBV-infected HepaRG cells by anti-S PNA coupled to Lactose increases its uptake and decreases HBsAg release [37].

### 6.2. Lipid-Oligonucleotide Conjugates

Similarly, lipids represent another delivery system which is used to facilitate oligonucleotide entry into the cells. Several lipid-based systems for oligonuleotide delivery have been studied. Thus, cholesterol has been used largely to improve siRNA uptake. Interestingly, cholesterol-siRNA conjugates can reach specific tissues and inhibit gene expression [59]. Other lipid-based-systems have been used for chronic HBV treatment and greatly increase siRNA activity when compared to uncoupled siRNA molecules [59,60].

### 6.3. Polymer-Oligonucleotide Conjugates

Polymers are also among molecules that are used as strategy in intracellular uptake of oligonucleotides. Moreover, Polymers used as vehicles protect their cargos against nuclease degradation. In addition, the combination between negatively charged oligonucleotides and cationic Polymers leads to formation of polyplexes which favor cellular uptake of molecule [60]. Thus, these electrostatic properties allow polymer-based carriers to improve intracellular internalization of oligonucleotides such as siRNA, consequently these polyplexes increase the gene silencing [59].

### 6.4. Aptamer-Oligonucleotide Conjugates

Aptamers are synthetic oligonucleotides of 20 to 80 bases that are able to bind to specific molecules such as siRNA with great affinity. In addition, aptamers are able to recognize cell surface receptors in order to mediate targeted delivery of bioactive molecules both in vitro and in vivo [60]. Thus, aptamers can to be used as vehicles to delivery antisense molecules such as siRNA and PNA into the cells to control gene expression by their silencing. Interestingly, there are several approaches for coupling oligonucleotides with aptamers. Indeed, aptamers can be directly coupled to oligonucleotide terminus or by using a linker moiety between both molecules. Multiples studies are described and show an advantage of aptamer-based transporters in therapeutic drug delivery [30,59,61].

## 7. Conclusions

Altogether, these results established that several new drugs such as PNAs and siRNA conjugated to different molecules such as CPPs, which represent the powerful tools for the development of the efficient therapies against chronic Hepatitis B virus infection. In addition, our results provide the first evidence that PNA targeting viral epsilon sequence and conjugated to CPPs inhibit Hepadnavirus replication in vitro and in vivo in duck HBV (DHBV) model. Interestingly, some CPPs alone such as (D-Arg)_8_ and its modified form termed Decanoyl-(D-Arg)_8_ are able to inhibit viral replication without toxicity in cell cultures. Importantly, our results suggest that the choice of CPP used as vehicles for intracellular uptake of some oligonucleotides such as PNAs may play an essential role in the specificity of this inhibition. Thus, these data indicate that CPP-siRNAs, CPP-PNAs, CPPs, and modified CPPs appear as a promising therapeutic approach for chronic hepatitis B treatment. In order to achieve clearance of chronic infection, it would be relevant to combine these new drugs with current treatments or use CPP-siRNAs combined to CPP-PNAs in bi-therapy. Indeed, this therapeutic strategy could represent a potent anti-HBV treatment.

## Figures and Tables

**Table 1:** Different therapeutic drugs and their biological activities against hepatitis B virus.

Drugs	Gene Delivery System	Target	Cells/Animal Models	Antiviral Effect	Toxicity
PNA alone	-	Duck HBV	PDH cell cultures	+	− [9]
CPP-PNA	CPP	Duck HBV	PDH cell cultures Ducklings	+	− [9,12]
siRNA alone	-	HBV	Huh-7 cells Transgenic mouse HepG2.2.15	+	− [25,26,27]
CPP-siRNA	CPP	HBV	Transgenic mouse HepG2.2.15	+	− [13,28,48]
CPP	-	Duck HBV	PDH cell cultures Ducklings	+	− [12]
Modified CPP	-	Duck HBV HBV	PDH cell cultures HepG2.2.15 cells	+	− [53]
